# [^68^Ga]Ga-HER2 Affibody PET/CT for early prediction of neoadjuvant therapy outcome in HER2-positive breast cancer: a preliminary report

**DOI:** 10.1186/s13550-026-01424-w

**Published:** 2026-04-02

**Authors:** Yuhan Sun, Ran An, Ruoxi Yang, Xiao Pang, Xiaoshan Chen, Mengjiao Wang, Jianqiang Zhao, Xiaolin Chen, Qiang Fu, Yunuan Liu, Jingya Han, Na Wang, Yan Zhao, Xinming Zhao

**Affiliations:** 1 Department of Nuclear Medicine, The Fourth Hospital of Hebei Medical University, 12 Jiankang Road, Shijiazhuang, 050011 Hebei China; 2 Department of Oncology, The Fourth Hospital of Hebei Medical University, 12 Jiankang Road, Shijiazhuang, 050011 Hebei China; 3 Hebei Provincial Key Laboratory of Tumor Microenvironment and Drug Resistance, Shijiazhuang, 050011 Hebei China

**Keywords:** Breast cancer, HER2, PET/CT, Pathological complete response, Neoadjuvant therapy

## Abstract

**Background:**

Neoadjuvant therapy (NAT) has become a standard treatment for HER2-positive breast cancer. However, patient responses vary substantially, and reliable methods for early response assessment are still lacking. This study aimed to evaluate the value of [^68^Ga]Ga-HER2 Affibody PET/CT for the early assessment of treatment outcome by predicting pathological complete response (pCR) in HER2-positive breast cancer.

**Results:**

32 of the 54 enrolled patients achieved pCR (59.3%). Following two NAT cycles, [^68^Ga]Ga-HER2 Affibody PET/CT parameters decreased from baseline in all patients (*P* < 0.001). Early percentage changes in PET parameters (ΔSUV%, ΔSUL%, ΔTLA%, ΔTBR%) and their absolute values after the second NAT cycle were associated with pCR (r range: -0.658 to -0.273; *P <* 0.05). ΔTBR% demonstrated the best predictive value for pCR (AUC = 0.918), with 93.8% sensitivity and 86.4% specificity at a cutoff of -70.5%. In contrast, tumor size assessment based on RECIST 1.1 showed lower predictive performance, with a sensitivity of 56.3% (18/32) and a specificity of 45.5% (10/22).

**Conclusion:**

This study demonstrates the potential of [^68^Ga]Ga-HER2 Affibody PET/CT to predict NAT outcome early in HER2-positive breast cancer, which could facilitate subsequent treatment optimization.

**Supplementary Information:**

The online version contains supplementary material available at 10.1186/s13550-026-01424-w.

## Introduction

 According to the latest (2022) global cancer figures from GLOBOCAN, breast cancer is the second most commonly diagnosed cancer worldwide and the leading cause of cancer-related death in women [1]. Human epidermal growth factor receptor 2 (HER2) is overexpressed in approximately 20–25% of newly diagnosed breast cancer cases, which correlates with a poor prognosis and reduced overall survival [2]. For these patients, neoadjuvant therapy (NAT) with dual anti-HER2 agents and chemotherapy constitutes the standard of care, reducing the surgical extent while improving survival and quality of life [3].

Given the high cost and significant variation in the individual efficacy of NAT, the early identification of patients likely to benefit from it is critical [4]. Pathological complete response (pCR) is the most critical and objective endpoint for assessing NAT efficacy and is recognized as a strong prognostic factor for predicting both disease-free and overall survival in patients with breast cancer [5]. Therefore, early and accurate prediction of pCR is essential for formulating subsequent treatment strategies [6]. Currently, however, pCR confirmation relies entirely on histopathological assessment of surgical specimens obtained after treatment completion, with no recognized evaluation method available for accurate prediction in the early treatment course (after two or four courses of treatment) [7]. Although advanced imaging techniques, such as MRI, are valuable for assessing pathological response after NAT for breast cancer, their predictive accuracy varies across studies [8–9]. Therefore, developing methods that can reliably predict pCR early in the treatment process is of great clinical importance.

PET/CT employs radiolabeled targeted probes to image specific biochemical processes, enabling non-invasive and dynamic quantification of tumor morphology and function [10]. The conventional tracer ^18^F-fluorodeoxyglucose ([^18^F]F-FDG) reflects tumor metabolic activity through its accumulation; however, its utility in the routine diagnosis of primary breast cancer is constrained by tumor size and variations in biological characteristics, including histological subtype, tumor grade, and hormone receptor status [11]. In contrast, HER2-targeted molecular probes are designed to specifically bind to HER2 receptors on the tumor cell surface, enabling precise assessment of HER2 expression across systemic lesions [12–13]. Notably, in HER2-positive primary lesions, both the maximum standardized uptake value and the target-to-background ratio were higher for HER2-targeted molecular probes than for [^18^F]F-FDG [14]. Among available targeting strategies, the Affibody molecular scaffold offers distinct advantages, including high binding affinity and low molecular weight (approximately 6.5 kDa), which facilitate rapid plasma clearance [15]. This favorable pharmacokinetic profile effectively circumvents the slow clearance and high radiation exposure typically associated with antibody-based tracers such as trastuzumab [16]. Preclinical data further support the favorable tolerability and safety profile, which underscores its translational potential [17]. However, the clinical utility of [^68^Ga]Ga-HER2 Affibody PET/CT in predicting the efficacy of NAT remains to be elucidated.

This study aimed to evaluate the ability of [^68^Ga]Ga-HER2 Affibody PET/CT to predict early the pathological response to NAT and assess treatment outcome in patients with HER2-positive breast cancer, particularly after two cycles. These findings are expected to provide a foundation for the optimization of therapeutic strategies.

## Materials and methods

### Patients and study design

This single-center study was approved by the institutional ethics committee (Approval No. 2022054). Consecutively enrolled patients provided written informed consent between June 2023 and August 2025.

The inclusion criteria were as follows: (1) women aged 18–75 years; (2) biopsy-confirmed HER2-positive breast cancer, defined as an immunohistochemistry (IHC) score of 3 + or 2 + with a positive fluorescence in situ hybridization (FISH) result, in accordance with the American Society of Clinical Oncology (ASCO) guidelines [18]; (3) no prior treatment before baseline [^68^Ga]Ga-HER2 Affibody PET/CT; (4) all patients underwent at least two protocol-defined PET/CT scans: PET 1 (baseline) and PET 2 (after completing two cycles of NAT); (5) patients who met the indications for NAT for breast cancer, completed the full NAT course, and subsequently underwent surgical resection. The exclusion criteria included the following: (1) HER2-low-expressing or HER2-negative breast cancer; (2) concurrent other malignancies; (3) unwillingness to undergo the protocol-specified PET/CT imaging; (4) PET/CT performed outside the predefined study time windows; (5) evidence of distant metastasis after examination.

### Treatment schemes

NAT was administered according to established guidelines [19]. All enrolled patients with HER2-positive breast cancer completed the full NAT course. The treatment backbone consistently consisted of dual HER2 blockade combined with chemotherapy. Variations in the specific regimens arose from individualized adaptations of the chemotherapy components and other targeted agents, based on distinct clinical indications. The distribution of the specific neoadjuvant regimens in this cohort was as follows: TCbHP (nab-paclitaxel, carboplatin, trastuzumab, and pertuzumab) in 16 patients; AC-TH (doxorubicin, cyclophosphamide, nab-paclitaxel, trastuzumab, and pertuzumab) plus pyrotinib in 23 patients; THP (nab-paclitaxel, trastuzumab, and pertuzumab) in 6 patients; TH (nab-paclitaxel and trastuzumab) plus pyrotinib in 3 patients; and TCbH (nab-paclitaxel, carboplatin, and trastuzumab) plus pyrotinib in 6 patients.

### PET/CT imaging

[^68^Ga]Ga-HER2 Affibody (111–185 MBq) was intravenously administered via the antecubital vein. PET/CT imaging was initiated 50 min post-injection using a Vereos PET/CT scanner (Philips, The Netherlands). All patients were positioned supine, with their arms elevated above their heads. First, a spiral CT scan was performed from the head to the thigh base using a standard-dose protocol with the following parameters: tube voltage, 120 kV; tube current, 60 mA; pitch, 0.813; matrix, 768 × 768; slice thickness, 4 mm; and field of view (FOV) 600 mm. Immediately thereafter, PET data were acquired in three-dimensional mode at 1.5 min per bed position, with a matrix of 144 × 144, slice thickness of 3 mm, and a FOV of 576 mm. Images were reconstructed using the Ordered Subset Expectation Maximization (OSEM) algorithm with CT-based attenuation correction. All datasets (1024 × 1024 pixels) were transferred to a dedicated IntelliSpace Portal workstation for further processing and analyses. The detailed molecular structure of the radiotracer is provided in Supplementary File 1.

### Image analysis

Serial PET/CT scans were independently evaluated by two experienced nuclear medicine physicians who were blinded to all clinical and therapeutic information. Discrepancies between the two initial readers were adjudicated by a third senior nuclear medicine physician. Image-positive lesions were defined as those with tracer uptake higher than the adjacent background level that did not correspond to physiologic or normal variants of the tracers, and CT scans were utilized for correlation to eliminate nonspecific findings. For the primary breast tumor, the maximum, mean, and peak standardized uptake values (SUVmax, SUVmean, and SUVpeak) and their lean body mass-normalized counterparts (SULmax, SULmean, and SULpeak) were first determined. The [^68^Ga]Ga-HER2 Affibody-avid tumor volume (HTV) was subsequently delineated automatically on the PET images by applying a threshold of 40% of the SUVmax. We also derived the total lesion activity (TLA), which was calculated as the SUVmean × HTV. For lesions demonstrating low or absent radiotracer uptake, boundaries were manually defined with reference to the corresponding CT anatomy. For axillary metastatic lymph nodes (MLN), when multiple involved nodes were present, the one with the highest uptake was selected, and its SUVmax was measured. To calculate the tumor-to-background ratio (TBR), we placed a 10-mm spherical ROI in the descending aorta, measured the mean standardized uptake value of the mediastinal blood pool (SUVmean-mbp), and then divided the tumor SUVmax by this value.

Tumor size was assessed based on the sum of the longest diameters (SLD) of the target lesions according to RECIST 1.1 criteria [20]. Target lesions were identified on contrast-enhanced CT or MRI scans obtained from routine clinical records. For a single lesion, the long-axis diameter on the most prominent slice was measured. In multifocal disease, the SLD was calculated as the sum of the diameters of all individual foci.

All parameters were measured at baseline and post-treatment, and the percentage change (Δ%) calculated as follows: [(post-value - pre-value) / pre-value] × 100%.

### Surgery and pathological assessment

All 54 patients underwent surgical resection, which included mastectomy (*n* = 47) and breast-conserving surgery (*n* = 7). Axillary management consisted of sentinel lymph node biopsy (SLNB) alone (*n* = 7), upfront axillary lymph node dissection (ALND) (*n* = 46), or completion ALND following SLNB-confirmed metastasis (*n* = 1).

pCR was defined as the absence of invasive carcinoma in the breast and axillary lymph nodes after completion of neoadjuvant therapy, allowing for the presence of ductal carcinoma in situ (ypT0/is) [21]. Patients who did not meet this criterion were classified as non-pCR, and the extent of residual disease in these cases was further quantified using the Residual Cancer Burden (RCB) system [22].

### Statistical analysis

Statistical analyses were performed using IBM SPSS Statistics software (version 25.0). Continuous variables are expressed as mean ± standard deviation or median (interquartile range), based on their distribution. Categorical variables are presented as numbers (percentages). Differences in [^68^Ga]Ga-HER2 Affibody PET/CT parameters and tumor size measurement between pCR and non-pCR groups were compared using the Mann-Whitney U test. Associations among PET/CT parameters, lesion size, and pCR status were assessed with Spearman’s correlation. Receiver operating characteristic (ROC) analysis was used to evaluate the predictive performance of each parameter for pCR, with the optimal cutoff values determined by the Youden index. The areas under the curves (AUC) were compared using DeLong’s test. All tests were two-sided, with *P* < 0.05 considered statistically significant. No adjustment for multiple comparisons was made because of the exploratory nature of the study design.

## Results

### Patient characteristics

**Fig. 1:**
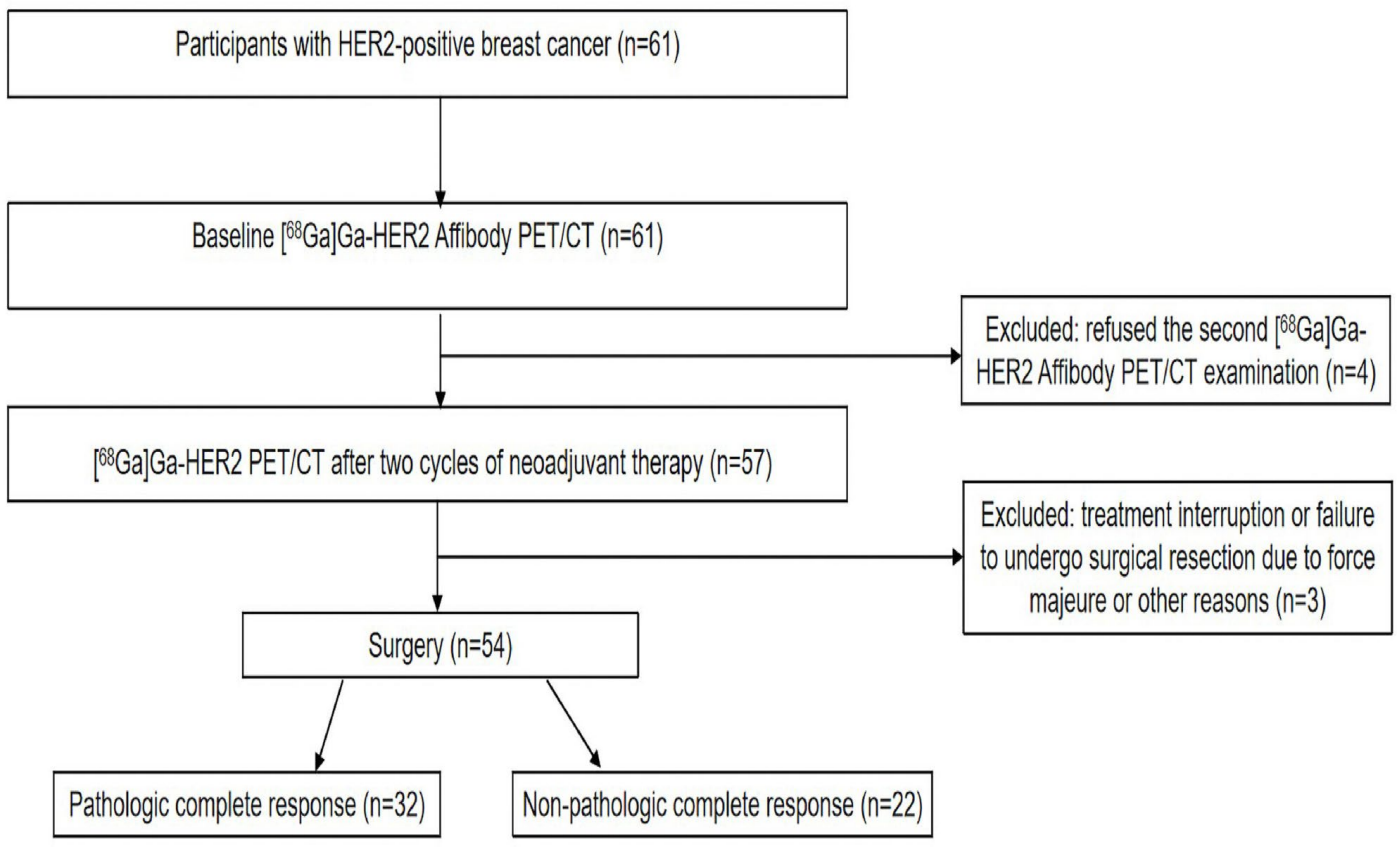
Flowchart of the study population

A total of 54 patients were included in the final analysis. The mean age of the study population was 50.96 ± 1.47 years. pCR was achieved in 32 patients (59.3%). A flowchart of the study design is presented in Fig. 1. The baseline clinical characteristics were comparable between patients who achieved pCR and those who did not (Table 1). The baseline scan (PET1) was performed 2.33 ± 1.59 days before NAT initiation, and the follow-up scan (PET2) was conducted 18.20 ± 4.49 days after two NAT cycles.

**Table 1:** Clinical and pathological characteristics of patients

Characteristic	Patients		pCR	non-pCR	*P* value
	*n* = 54		*n*=32	*n*=22	
Age (y)	50.96 ± 1.47		50.91 ± 0.273	51.05 ± 0.283	0.826
Menstrual state					1.0
Premenopausal	27(50%)		16(59.3%)	11(40.7%)	
Postmenopausal	27(50%)		16(59.3%)	11(40.7%)	
Pre-NAT pathological tumor staging					0.641
ypT1	11(20.4%)		8(72.7%)	3(27.3%)	
ypT2	31(57.4%)		16(51.6%)	15(48.4%)	
ypT3	5(9.2%)		4(80%)	1(20%)	
ypT4	7(13.0%)		4(57.1%)	3(42.9%)	
Pre-NAT pathological lymph node staging					0.976
ypN0	8(14.8%)		4(50%)	4(50%)	
ypN1	32(59.3%)		21(65.6%)	11(34.4%)	
ypN2	6(11.1%)		3(50%)	3(50%)	
ypN3	8(14.8%)		4(50%)	4(50%)	
Pre-NAT AJCC clinical stage					0.899
IIA	18(33.3%)		11(61.1%)	7(38.9%)	
IIB	12(22.2%)		7(58.3%)	5(41.7%)	
IIIA	7(13.0%)		4(57.1%)	3(42.9%)	
IIIB	7(13.0%)		5(71.4%)	2(28.6%)	
IIIC	10(18.5%)		5(50%)	5(50%)	
Histological type					0.141
Invasive Ductal Carcinoma	50 (92.6%)		31(62.0%)	19(38.0%)	
Invasive Lobular Carcinoma	2 (3.7%)		1(50%)	1(50%)	
Other Special Type	2 (3.7%)		1(50%)	1(50%)	
Primary tumor size (cm)					0.641
SLD	3.32(2.43,4.21)		3.34(1.94,4.74)	3.98(2.47,5.49)	

Data are numbers of patients, with percentages in parentheses, or medians ± SDs

AJCC, American Joint Commission on Cancer; pCR, pathological complete response; SLD, the sum of the longest diameters; NAT, neoadjuvant therapy

### Changes in [^68^Ga]Ga-HER2 Affibody PET/CT and tumor size parameters from baseline to after two cycles of NAT

The [^68^Ga]Ga-HER2 Affibody PET/CT and tumor size parameters are summarized in Table 2 for the two time points: baseline (PET1) and after two cycles of NAT (PET2). A comparison of the median values revealed significant declines in all measured parameters (including SUVmax, SUVpeak, SUVmean, SULmax, SULpeak, SULmean, HTV, TLA, TBR, MLN SUVmax, and SLD) following treatment (all *P <* 0.001).

**Table 2:** Changes in [^68^Ga]Ga-HER2 Affibody PET/CT and tumor size parameters from baseline to after two cycles of NAT

Parameters	Baseline PET1	After two cycles of NAT PET2	*P* value
SUVmax	3.25(2.34,4.17)	0.94(0.56,1.51)	< 0.001
SUVmean	1.94(1.39,2.58)	0.54(0.41,0.91)	< 0.001
SUVpeak	2.63(1.86,3.12)	0.69(0.48,1.15)	< 0.001
SULmax	2.21(1.69,2.77)	0.63(0.40,1.04)	< 0.001
SULmean	1.34(1.04,1.60)	0.37(0.28,0.65)	< 0.001
SULpeak	1.85(1.32,2.27)	0.47(0.34,0.81)	< 0.001
HTV	10.85(5.17,20.45)	4.45(1.89,10.32)	< 0.001
TLA	25.00(7.83,50.64)	2.92(0.84,8.12)	< 0.001
TBR	1.53(0.96,2.49)	0.29(0.16,0.53)	< 0.001
MLN SUVmax	2.57(1.78,3.83)	0.73(0.52,1.06)	< 0.001
SLD	3.55(2.00,5.43)	2.06(1.18,3.75)	< 0.001

SUVmax, standardized uptake value; SUVmean, mean standardized uptake value; SUVpeak, peak standardized uptake value; SULmax, lean body mass–normalized maximum standardized uptake value; SULmean, lean body mass–normalized mean standardized uptake value; SULpeak, lean body mass–normalized peak standardized uptake value; HTV, [^68^Ga]Ga-HER2 affibody-avid tumour volume; TLA, total lesion activity; TBR, tumor-to-background ratio; MLN, metastatic lymph node; SLD, sum of the longest diameters; NAT, neoadjuvant therapy

### Comparison of [^68^Ga]Ga-HER2 Affibody PET/CT with tumor size parameters by NAT response

Based on postoperative pathological findings, patients were stratified into pCR and non-pCR groups. The comparison of [^68^Ga]Ga-HER2 Affibody PET/CT and tumor size parameters between the pCR and non-pCR patients is summarized in Table 3. At baseline, no significant differences in [^68^Ga]Ga-HER2 Affibody PET/CT or tumor size parameters were detected between the groups (all *P* > 0.05). After two cycles of NAT, the Post-SUVmax, Post-SUVmean, Post-SUVpeak, Post-SULmax, Post-SULmean, Post-SULpeak, Post-TLA, and Post-TBR values were all lower in the pCR group than in the non-pCR group (all *P* < 0.05). The percentage reductions of [^68^Ga]Ga-HER2 Affibody PET/CT parameters (including ΔSUVmax%, ΔSUVmean%, ΔSUVpeak%, ΔSULmax%, ΔSULmean%, ΔSULpeak%, ΔTLA%, and ΔTBR%) were greater in the pCR group than in the non-pCR group (all *P* < 0.05).

**Table 3:** Comparison of [^68^Ga]Ga-HER2 Affibody PET/CT with tumor size parameters by NAT response

Parameters	pCR	non-pCR	*P* value *	*r*	*P* value †
	*n* = 32	*n* = 22			
Pre-SUVmax	3.43(2.69,4.14)	3.13(2.23,4.33)	0.487	0.032	0.819
Pre-SUVmean	2.02(1.62,2.47)	1.86(1.33,2.69)	0.509	0.027	0.849
Pre-SUVpeak	2.72(2.06,3.19)	2.42(1.60,3.72)	0.398	0.039	0.777
Pre-SULmax	2.36(1.80,2.83)	2.08(1.52,2.88)	0.398	0.018	0.897
Pre-SULmean	1.40(1.08,1.59)	1.19(0.95,1.85)	0.444	0.033	0.812
Pre-SULpeak	1.92(1.39,2.25)	1.53(1.23,2.76)	0.360	0.025	0.858
Pre-HTV	9.86(5.03,20.75)	12.89(4.90,25.76)	0.311	0.120	0.385
Pre-TLA	24.79 (7.26,50.11)	27.36(9.11,66.37)	0.597	0.170	0.219
Pre-TBR	1.73 (1.07,3.07)	1.13(0.91,2.09)	0.081	0.219	0.112
Pre-MLNSUVmax	2.57(1.68,4.15)	2.64(1.82,3.36)	0.874	0.053	0.701
Pre-SLD (cm)	3.25(1.90,4.50)	3.75(2.40,5.93)	0.172	0.192	0.163
Post-SUVmax	0.82(0.51,1.22)	1.45(0.79,3.51)	0.004	-0.437	0.001
Post-SUVmean	0.46(0.32,0.68)	0.87(0.45,2.19)	0.006	-0.436	0.001
Post-SUVpeak	0.63(0.40,0.92)	1.07(0.57,2.01)	0.004	-0.417	0.002
Post-SULmax	0.55(0.35,0.89)	0.97(0.53,2.34)	0.006	-0.431	0.001
Post-SULmean	0.32(0.25,0.45)	0.54(0.32,1.42)	0.004	-0.443	0.002
Post-SULpeak	0.42(0.28,0.59)	0.72(0.39,1.35)	0.005	-0.413	0.002
Post-HTV	3.74(1.58,10.35)	5.40(2.77,10.44)	0.107	-0.185	0.180
Post-TLA	1.62(0.62,6.04)	3.93(1.40,15.78)	0.007	-0.273	0.046
Post-TBR	0.21(0.16,0.34)	0.46(0.33,1.19)	0.001	-0.507	< 0.001
Post-MLNSUVmax	0.70(0.53,0.88)	0.77 (0.52,1.20)	0.181	-0.193	0.162
Post-SLD (cm)	1.95(1.06,3.05)	2.19(1.20,3.55)	0.252	-0.167	0.227
ΔSUVmax%	-78.00(-86.00,-59.50)	-54.00(-67.75,-27.75)	< 0.001	-0.472	< 0.001
ΔSUVmean%	-77.00(-84.75,-50.75)	-50.05(-66.75,-30.50)	0.001	-0.448	0.001
ΔSUVpeak%	-79.00(-86.75,-60.05)	-55.00(-66.11,-35.63)	< 0.001	-0.484	< 0.001
ΔSULmax%	-78.00(-85.75,-59.25)	-54.50(-71.25,-27.75)	0.001	-0.450	0.001
ΔSULmean%	-76.05(-87.75,-60.00)	-50.00 (-69.25,-30.00)	0.001	-0.449	0.001
ΔSULpeak%	-79.50(-85.25,-59.00)	-54.00(-68.25,-33.00)	< 0.001	-0.472	< 0.001
ΔHTV%	-51.00(-67.25,-37.50)	-48.50(-77.25,-26.50)	0.603	-0.012	0.879
ΔTLA%	-88.50(-95.00,-84.25)	-77.00(-87.00,-51.50)	0.001	-0.318	0.019
ΔTBR%	-87.50(-94.00,-79.25)	-59.50(-63.96,-41.24)	< 0.001	-0.658	< 0.001
ΔMLN SUVmax%	-73.50(-88.64,-46.50)	-53.58(-68.50,-45.50)	0.088	-0.208	0.132
ΔSLD%	-41.41(-48.33,-20.78)	-37.88(-48.30,-10.93)	0.660	-0.063	0.651

* *P* values, Mann-Whitney *U* test; †, Spearman’s rank correlation test. Abbreviations: pCR, pathological complete response; Pre-, before neoadjuvant therapy; Post-, after two cycles of neoadjuvant therapy; r, point-biserial correlation coefficient; SUVmax, standardized uptake value; SUVmean, mean standardized uptake value; SUVpeak, peak standardized uptake value; SULmax, lean body mass–normalized maximum standardized uptake value; SULmean, lean body mass–normalized mean standardized uptake value; SULpeak, lean body mass–normalized peak standardized uptake value; HTV, [^68^Ga]Ga-HER2 affibody-avid tumor volume; TLA, total lesion activity; TBR, tumour-to-background ratio; MLN, metastatic lymph node; SLD, sum of the longest diameters

The post-treatment values of HTV, MLN SUVmax, and SLD showed no significant differences between the pCR and non-pCR groups. Similarly, the percentage reductions of these parameters (ΔHTV%, ΔMLN SUVmax%, and ΔSLD%) showed no statistically significant differences between the two groups (all *P* > 0.05).

### Association of [^68^Ga]Ga-HER2 Affibody PET/CT and tumor size parameters with pathological response

Following two cycles of NAT, significant correlations were observed between pCR and [^68^Ga]Ga-HER2 Affibody PET/CT parameters, including Post-SUVmax, Post-SUVmean, Post-SUVpeak, Post-SULmax, Post-SULmean, Post-SULpeak, Post-TLA, and Post-TBR (*r* = -0.437, -0.436, -0.417, -0.431, -0.443, -0.413, -0.273, -0.507; *P* = 0.001, *P* = 0.001, *P* = 0.002, *P* = 0.001, *P* = 0.002, *P* = 0.002, *P* = 0.046, and *P <* 0.001 respectively). Similarly, percentage reductions in [^68^Ga]Ga-HER2 Affibody PET/CT parameters from baseline to post-NAT, specifically ΔSUVmax%, ΔSUVmean%, ΔSUVpeak%, ΔSULmax%, ΔSULmean%, ΔSULpeak%, ΔTLA%, and ΔTBR%, correlated with pCR (*r* = -0.472, -0.448, -0.484, -0.450, -0.449, -0.472, -0.318, -0.658; *P* < 0.001, *P* = 0.001, *P* < 0.001, *P* = 0.001, *P* = 0.001, *P* < 0.001, *P* = 0.02, and *P <* 0.001).

**Fig. 2:**
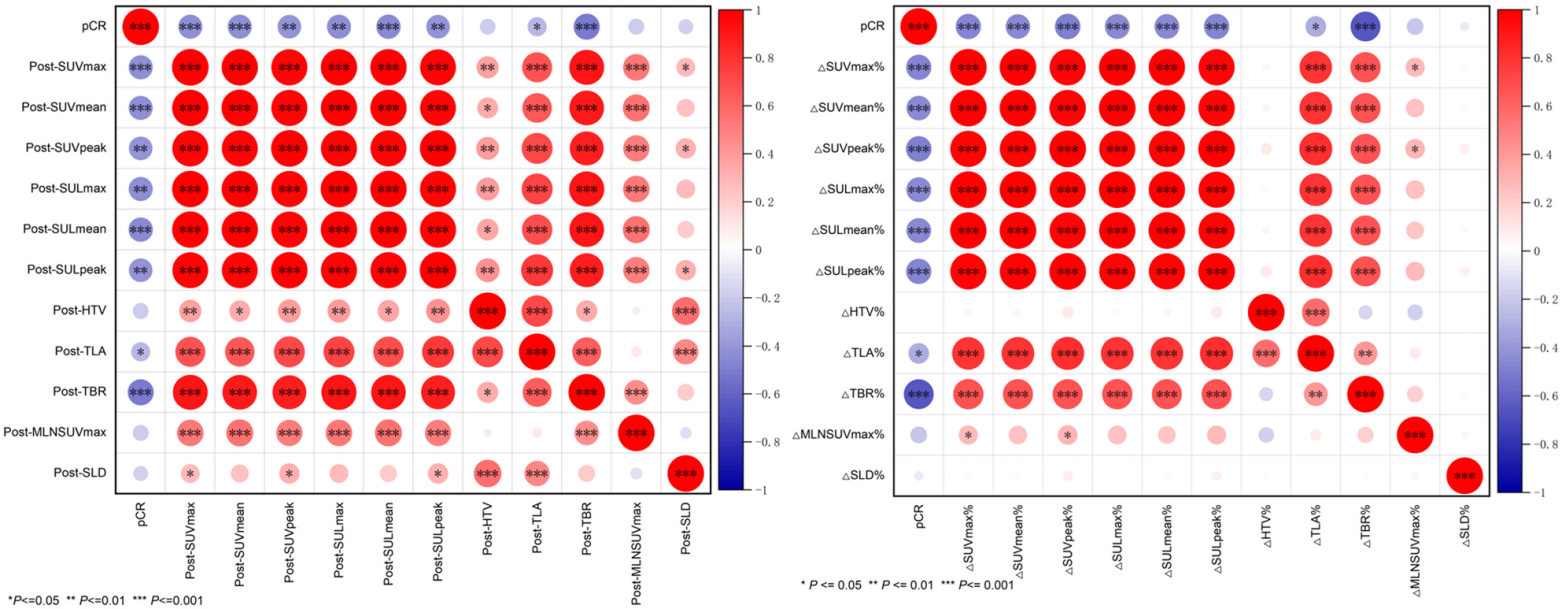
Association of [^68^Ga]Ga-HER2 Affibody PET/CT and tumor size parameters with pathological response. Red circles indicate positive correlations, while blue circles represent negative correlations. Darker shades denote stronger correlation magnitudes. *P* values reflect the statistical significance of the correlations. * *P* < 0.05. ** *P* < 0.01. *** *P* < 0.001

The post-treatment values of HTV, MLN SUVmax, and SLD (*r* = -0.185, -0.193, -0.167; *P* = 0.180, *P* = 0.162, *P* = 0.227), as well as the percentage reductions of these parameters (ΔHTV, ΔMLN SUVmax%, and ΔSLD%) (*r* = -0.012, -0.208, -0.063; *P* = 0.879, *P* = 0.132, *P* = 0.651) showed no significant correlation with pCR (Fig. 2; Table 3).

### Predictive performance of [^68^Ga]Ga-HER2 Affibody PET/CT for pathological response

**Fig. 3:**
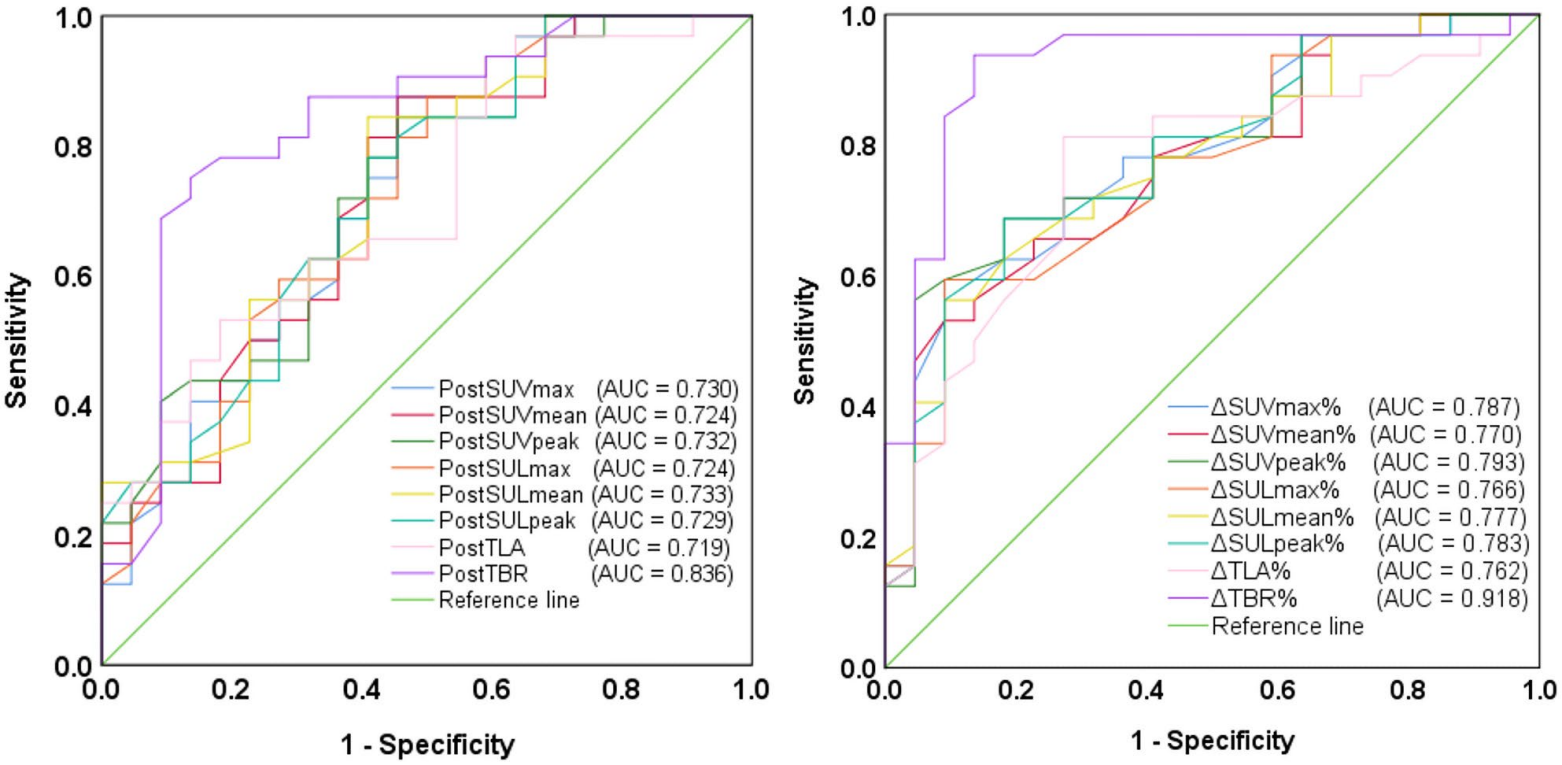
Receiver operating characteristic curves evaluating early predictive accuracy of [^68^Ga]Ga-HER2 Affibody PET/CT parameters for pathological response to neoadjuvant therapy

Receiver operating characteristic curve analysis was performed to evaluate the accuracy of [^68^Ga]Ga-HER2 Affibody PET/CT parameters in predicting pathological complete response and non-complete response following NAT in patients with HER2-positive breast cancer. The following parameters, measured after two cycles of NAT, were identified as significant predictors of pCR (Fig. 3): Post-treatment values: Post-SUVmax (AUC = 0.730), Post-SUVmean (AUC = 0.724), Post-SUVpeak (AUC = 0.732), Post-SULmax (AUC = 0.724), Post-SULmean (AUC = 0.733), Post-SULpeak (AUC = 0.729), Post-TLA (AUC = 0.719), Post-TBR (AUC = 0.836). Percentage changes from baseline (Δ%): ΔSUVmax% (AUC = 0.787), ΔSUVmean% (AUC = 0.770), ΔSUVpeak% (AUC = 0.793), ΔSULmax% (AUC = 0.766), ΔSULmean% (AUC = 0.777), ΔSULpeak% (AUC = 0.783), ΔTLA% (AUC = 0.762), ΔTBR% (AUC = 0.918).

The predictive performance of [^68^Ga]Ga-HER2 Affibody PET/CT for early pathological response to NAT is summarized in Table 4. ΔTBR% showed the highest accuracy for predicting pCR (AUC = 0.918, 95% CI: 0.833-1.000; *P <* 0.001), with 93.8% sensitivity and 86.4% specificity at the − 70.5% cutoff.

**Table 4:** Predictive performance of [^68^Ga]Ga-HER2 Affibody PET/CT for pathological response

ΔSUVmax%	AUC	Cutoff	Sensitivity (%)	Specificity (%)	95%CI	*P* value
	0.787	-74.0	59.4	90.9	0.665–0.909	< 0.001
ΔSUVmean%	0.770	-75.0	53.1	90.9	0.644–0.896	0.001
ΔSUVpeak%	0.793	-74.5	56.3	95.5	0.672–0.914	< 0.001
ΔSULmax%	0.766	-75.0	59.4	90.9	0.639–0.894	0.001
ΔSULmean%	0.777	-75.0	56.3	90.9	0.653–0.901	0.001
ΔSULpeak%	0.783	-69.5	68.8	81.8	0.660–0.907	< 0.001
ΔTLA%	0.762	-83.5	81.3	72.7	0.631–0.893	0.001
ΔTBR%	0.918	-70.5	93.8	86.4	0.833-1.00	< 0.001
Post-SUVmax	0.730	1.415	87.5	54.5	0.590–0.870	0.004
Post-SUVmean	0.724	0.835	87.5	54.5	0.583–0.864	0.006
Post-SUVpeak	0.732	0.995	84.4	54.5	0.596–0.868	0.004
Post-SULmax	0.724	1	87.5	50.0	0.583–0.865	0.006
Post-SULmean	0.733	0.52	84.4	59.1	0.595–0.871	0.004
Post-SULpeak	0.729	0.595	78.1	59.1	0.591–0.867	0.005
Post-TLA	0.719	10.72	93.8	40.9	0.582–0.856	0.007
Post-TBR	0.836	0.335	75.0	86.4	0.720–0.952	< 0.001

SUVmax, standardized uptake value; SUVmean, mean standardized uptake value; SUVpeak, peak standardized uptake value; SULmax, lean body mass–normalized maximum standardized uptake value; SULmean, lean body mass–normalized mean standardized uptake value; SULpeak, lean body mass–normalized peak standardized uptake value; TLA, total lesion activity; TBR, tumour-to-background ratio; AUC, area under curve

**Fig. 4:**
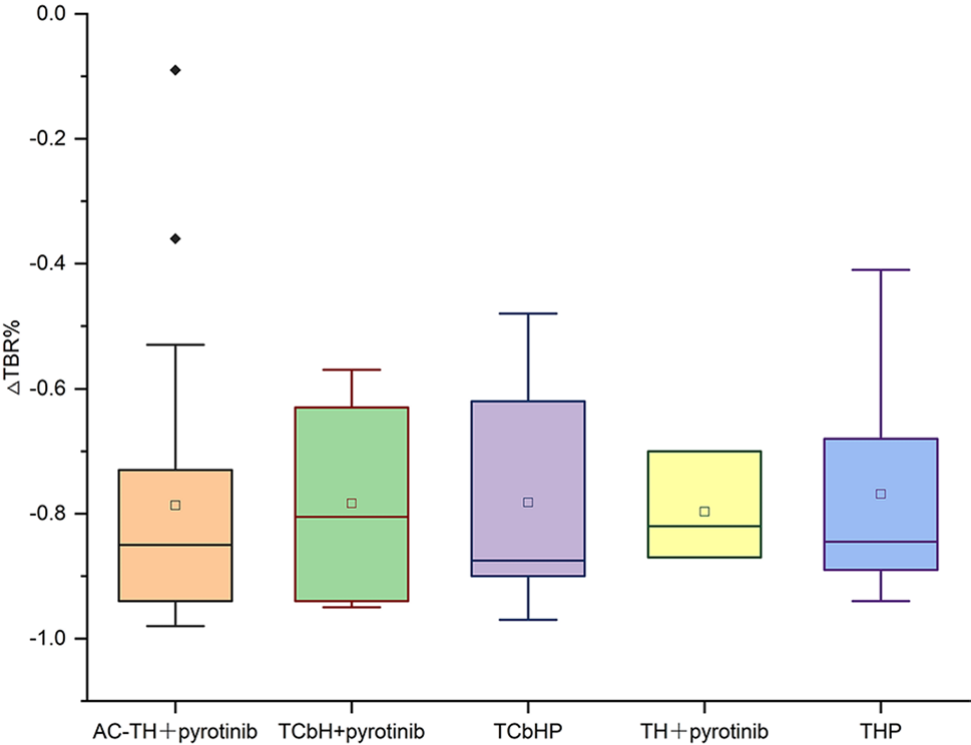
Comparison of ΔTBR% among neoadjuvant therapy regimens

The ΔTBR% of [^68^Ga]Ga-HER2 Affibody PET/CT was compared among the five NAT regimens. As shown in Fig. 4, no statistically significant differences in ΔTBR% were observed among the treatment groups.

**Fig. 5:**
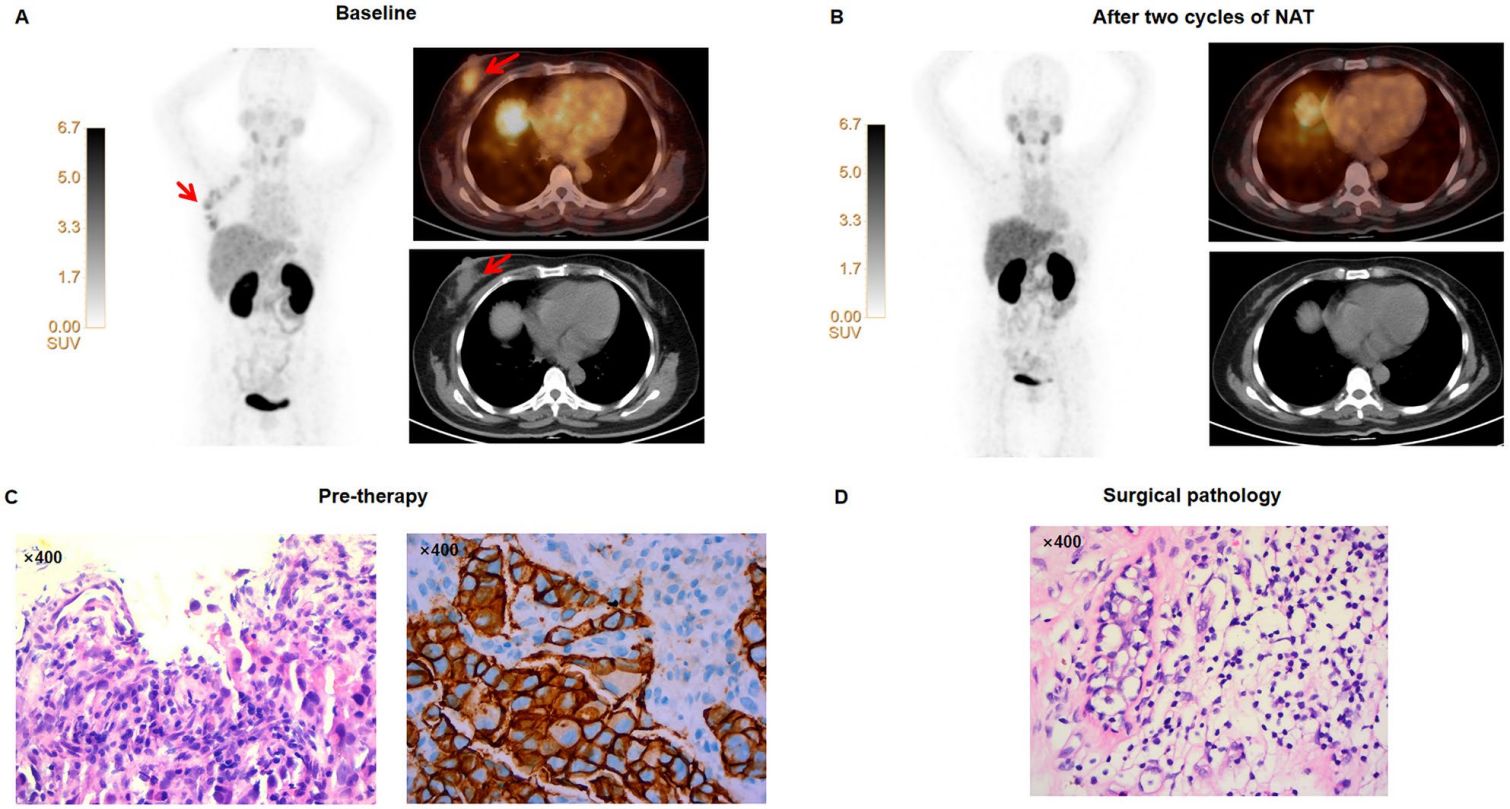
Representative [^68^Ga]Ga-HER2 PET/CT of a 64-year-old woman with left-sided invasive ductal carcinoma and who achieved pCR. (**A**) The baseline [^68^Ga]Ga-HER2 PET/CT scan shows a tumor lesion (red arrow; SUVmax, 3.22; TBR, 1.69). (**B**) After 2 NAT cycles, the [^68^Ga]Ga-HER2 PET/CT image demonstrates a marked decrease in tumor lesion radioactivity uptake (SUVmax, 1.05; TBR, 0.42). (**C**) Diagnostic biopsy of invasive ductal carcinoma shows tumor nests with nuclear atypia and cytoplasmic vacuolization. HER2 immunohistochemistry shows strong, complete membranous staining (IHC 3+), establishing the HER2-positive diagnosis. (**D**) The post-therapy resection specimen reveals only fibrotic stroma with no residual carcinoma, confirming a pathological complete response (pCR) to neoadjuvant chemotherapy

**Fig. 6:**
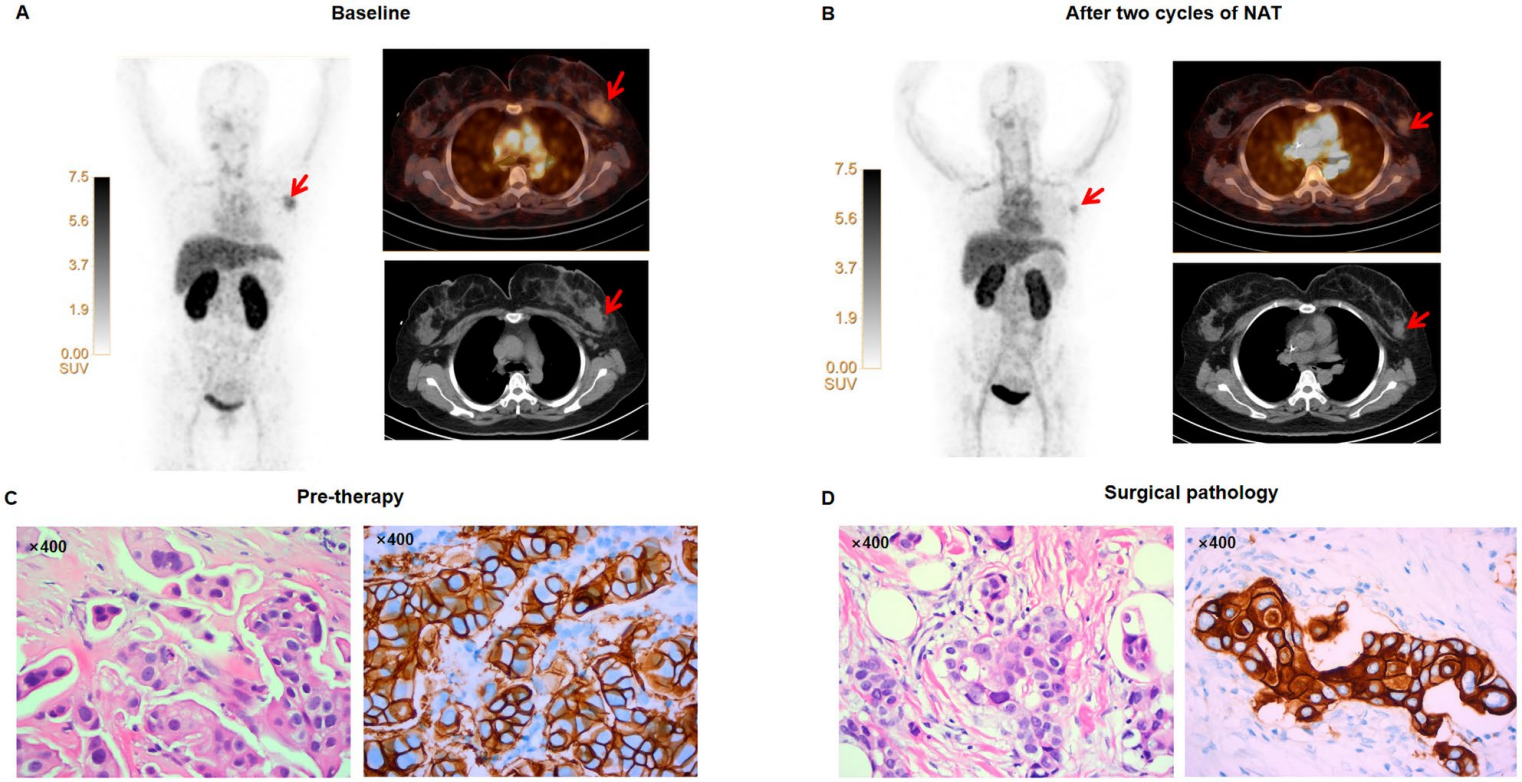
Representative [^68^Ga]Ga-HER2 PET/CT of a 58-year-old woman with right-sided invasive ductal carcinoma and who did not achieve pCR. (**A**) The baseline [^68^Ga]Ga-HER2 PET/CT scan shows a tumor lesion (red arrow; SUVmax, 4.26; TBR, 0.91). (**B**) After 2 NAT cycles, the [^68^Ga]Ga-HER2 PET/CT image demonstrates the tumor lesion with a minimal change in uptake (red arrow; SUVmax, 2.37; TBR, 0.37). (**C**) Diagnostic biopsy of invasive ductal carcinoma shows tumor nests with nuclear atypia and cytoplasmic vacuolization. HER2 immunohistochemistry shows strong, complete membranous staining (IHC 3+), confirming HER2 positivity. (**D**) The post-neoadjuvant therapy resection specimen shows a partial treatment response, featuring residual tumor nests amidst stromal fibrosis and lymphocytic infiltration. HER2 IHC confirms retained strong (3+) membranous expression

The baseline and post-treatment PET/CT images of representative pCR and non-pCR patients are shown in Figs. 5 and 6, respectively.

### Predictive performance of [^68^Ga]Ga-HER2 Affibody PET/CT versus tumor size parameters in pathological response

After two cycles of NAT, tumor size assessment based on RECIST 1.1 criteria demonstrated the following predictive performance for pathological response: sensitivity 56.3% (18/32), specificity 45.5% (10/22), PPV 60.0% (18/30), NPV 41.7% (10/24), and accuracy 51.9% (28/54) (Supplemental Table 1). In comparison, the optimal [^68^Ga]Ga-HER2 Affibody PET/CT parameter, ΔTBR%, exhibited higher predictive accuracy, achieving a sensitivity of 93.8% and a specificity of 86.4% at the optimal cutoff value of -70.5%, and an AUC of 0.918, as previously reported.

## Discussion

Neoadjuvant therapy (NAT) significantly improves the prognosis of patients with human epidermal growth factor receptor 2 (HER2)-positive breast cancer; however, interindividual variability in treatment response persists. Therefore, the early prediction of therapeutic efficacy and timely adjustment of treatment strategies are crucial for guiding clinical decision-making. This study evaluated the value of various PET-derived parameters in predicting pathological response to NAT and preliminarily demonstrated the clinical utility of [^68^Ga]Ga-HER2 Affibody PET/CT for the early prediction of treatment efficacy in patients with HER2-positive breast cancer. Specifically, after two cycles of neoadjuvant therapy, all patients showed a significant reduction in uptake compared to baseline, consistent with the treatment efficacy criteria defined by PERCIST 1.0 [23]. Importantly, patients with lower post-treatment uptake and greater reductions had a higher probability of achieving pathological complete response (pCR) in HER2-positive breast cancer.

Compared with previous HER2-targeted imaging studies using [^64^Cu]Cu-DOTA-trastuzumab or [^89^Zr]Zr-trastuzumab [24–25], this study not only confirms the significant association between early changes in PET/CT parameters and pCR in HER2-positive breast cancer, but also offers the key advantage of avoiding interference with ongoing anti-HER2 therapy during monitoring, since the Affibody molecule binds to a distinct HER2 epitope [26]. Building on this unique property, combined with the probe’s proven safety, rapid imaging capability, and excellent tissue penetration [27], [^68^Ga]Ga-HER2 Affibody PET/CT may enable dynamic monitoring of HER2 expression during therapy, facilitating early identification of patients with poor treatment response. The information thus obtained holds potential to inform the formulation of individualized treatment strategies. For example, if a patient shows only a small decrease in tracer uptake, treatment might be intensified or the chemotherapy regimen changed, with the goal of improving clinical outcomes and optimizing treatment plan.

Published guidelines recommend [^18^F]F-FDG PET/CT for staging high-risk breast cancer and monitoring HER2-targeted therapy response [28]. However, Guo et al. demonstrated that for HER2-positive breast cancer, HER2-targeted PET/CT exhibits significantly superior diagnostic performance compared to [^18^F]F-FDG, particularly in detecting primary tumors and metastatic lesions in lymph nodes, bone, and liver [14]. This study further demonstrated that, in contrast to the limited specificity of [^18^F]F-FDG PET/CT for pCR in HER2-positive breast cancer [29], the quantitative parameters derived from [^68^Ga]Ga-HER2 Affibody PET/CT exhibited robust predictive performance as early as after two cycles of NAT. Among these, ΔTBR% showed the highest diagnostic accuracy, with a reduction beyond − 70.5% indicating effective NAT and increased probability of pCR. In semi-quantitative assessments, TBR exhibited superior robustness against interference compared with SUVmax and SUVmean, more accurately reflecting the true biological distribution, thereby enhancing the precision of treatment response monitoring [30]. Moreover, a comparative analysis of ΔTBR% across the five treatment regimens revealed no significant intergroup differences, indicating that the early treatment response assessed using HER2-targeted PET is independent of the treatment regimen. Furthermore, unlike [^18^F]F-FDG, which requires an on-site cyclotron and on-demand synthesis, ^68^Ga can be consistently eluted from a generator with a long shelf life at a lower operational cost. This logistical advantage not only reduces dependency on centralized production facilities but also enhances the feasibility of implementing HER2-targeted PET/CT in resource-limited settings, thereby supporting its routine clinical utility in guiding NAT.

Previous studies have indicated that RECIST 1.1, while serving as a widely used imaging standard for evaluating treatment response in solid tumors, presents certain limitations when applied to breast cancer therapy [31]. Our findings demonstrate that although tumor size generally decreased after two cycles of NAT, imaging evaluation based on RECIST 1.1 criteria exhibited limited sensitivity and specificity for the early prediction of pCR. This observed constraint may be explained by several factors. Pathological alterations induced by chemotherapy, including reduced cellularity, necrosis, and fibrosis, do not consistently translate to measurable changes in overall tumor size [23]. Moreover, conventional unidimensional measurements fail to adequately characterize non-concentric regression patterns, such as the scattered and heterogeneous shrinkage seen in nest-like or dendritic morphological changes [32]. As a result, strict adherence to the RECIST criteria in this setting could lead to underestimation of the true treatment effect. Therefore, it is necessary to incorporate functional molecular information into the precision evaluation system to enhance the early identification of patients responsive to treatment, thereby providing a more reliable basis for guiding subsequent clinical decisions.

This study has several limitations. First, it was conducted at a single center with a limited sample size, and the findings require validation through multicenter studies. Second, this study exclusively evaluated the role of [^68^Ga]Ga-HER2 Affibody PET/CT in the early prediction of response to NAT in HER2-positive breast cancer, without concurrent [^18^F]F-FDG PET/CT examinations. Therefore, future head-to-head comparisons between these two tracers are warranted. Finally, due to varying clinical indications, the chemotherapy regimens in this cohort exhibited some heterogeneity. Future studies with larger sample sizes and more homogeneously treated cohorts are needed to validate our findings.

## Conclusion

This preliminary study indicates that [^68^Ga]Ga-HER2 Affibody PET/CT imaging can rapidly detect tumor changes after two cycles of neoadjuvant therapy, demonstrating its potential for the early prediction of treatment outcome in HER2-positive breast cancer. Future large-scale prospective studies are necessary to further validate the value of [^68^Ga]Ga-HER2 Affibody PET/CT in evaluating the efficacy of neoadjuvant therapy in HER2-positive breast cancer. 

## Supplementary Information

Below is the link to the electronic supplementary material.

Supplementary Material 1

## Data Availability

All data and materials are available from the corresponding authors upon reasonable request.

## Abbreviations

HER2: Human epidermal growth factor receptor 2
NAT: Neoadjuvant therapy
pCR: Pathological complete response
SLD: Sum of the longest diameters
SUVmax: Maximum standardized uptake value
SUVmean: Mean standardized uptake value
SUVpeak: Peak standardized uptake value
SULmax: Lean body mass–normalized maximum standardized uptake value
SULmean: Lean body mass–normalized mean standardized uptake value
SULpeak: Lean body mass–normalized peak standardized uptake value
HTV: [^68^Ga]Ga-HER2 affibody-avid tumour volume
TLA: Total lesion activity
TBR: Tumor-to-background ratio
MLN: Metastatic lymph node
AUC: Area under the curve
SLNB: Sentinel lymph node biopsy
ALND: Axillary lymph node dissection
H&E: Hematoxylin and eosin
