# Supplementary material for: [68Ga]Ga-HER2 Affibody PET/CT for early prediction of neoadjuvant therapy outcome in HER2-positive breast cancer: a preliminary report
Source: EJNMMI Res. 2026 Apr 2;16:73. doi: 10.1186/s13550-026-01424-w (PMC13168394; doi:10.1186/s13550-026-01424-w)
Supplement: Supplementary file 1 — Supplementary Material 1 [file 13550_2026_1424_MOESM1_ESM.docx]

Supplementary File 1: The sequence of [^68^Ga]Ga-HER2 affibody.

(DOTA-Ala-Glu-Asn-Lys-Phe-Asn-Lys-Glu-Met-Arg-Asn-Ala-Tyr-Trp-Glu-Ile-Ala-Leu-Leu-Pro-Asn-Leu-Asn-Asn-Gln-Gln-Lys-Arg-Ala-Phe-Ile-Arg-Ser-Leu-Tyr-Asp-Asp-Pro-Ser-Gln-Ser-Ala-Asn-Leu-Leu-Ala-Glu-Ala-Lys-Lys-Leu-Asn-Asp-Ala-Gln)

**Supplementary Table 1: Predictive Performance of Tumor Size Parameters for Pathologic Response**

| Criteria | pCR | non-pCR | Total |
| --- | --- | --- | --- |
| RECIST 1.1 |  |  |  |
| CR | 1 | 0 | 1 |
| PR | 17 | 12 | 29 |
| SD | 14 | 10 | 24 |
| PD | 0 | 0 | 0 |
| Total | 32 | 22 | 54 |

pCR = pathologic complete response; CR = complete response; PR = partial response; PD = progressive disease; SD = stable disease.
